# Roughness and Fiber Fraction Dominated Wetting of Electrospun Fiber-Based Porous Meshes

**DOI:** 10.3390/polym11010034

**Published:** 2018-12-27

**Authors:** Piotr K. Szewczyk, Daniel P. Ura, Sara Metwally, Joanna Knapczyk-Korczak, Marcin Gajek, Mateusz M. Marzec, Andrzej Bernasik, Urszula Stachewicz

**Affiliations:** 1 International Centre of Electron Microscopy for Materials Science, Faculty of Metals Engineering and Industrial Computer Science, AGH University of Science and Technology, 30-059 Kraków, Poland; pszew@agh.edu.pl (P.K.S.); urad@agh.edu.pl (D.P.U.); metwally@agh.edu.pl (S.M.); jknapczyk@agh.edu.pl (J.K.-K.); 2 Faculty of Materials Science and Ceramics, AGH University of Science and Technology, 30-059 Kraków, Poland; mgajek@agh.edu.pl; 3 Academic Centre for Materials and Nanotechnology, AGH University of Science and Technology, 30-059 Kraków, Poland; marzecm@agh.edu.pl (M.M.M.); bernasik@agh.edu.pl (A.B.); 4 Faculty of Physics and Applied Computer Science, AGH University of Science and Technology, 30-059 Kraków, Poland

**Keywords:** roughness, electrospinning, fiber, fraction, surface free energy, wetting, contact angle

## Abstract

Wettability of electrospun fibers is one of the key parameters in the biomedical and filtration industry. Within this comprehensive study of contact angles on three-dimensional (3D) meshes made of electrospun fibers and films, from seven types of polymers, we clearly indicated the importance of roughness analysis. Surface chemistry was analyzed with X-ray photoelectron microscopy (XPS) and it showed no significant difference between fibers and films, confirming that the hydrophobic properties of the surfaces can be enhanced by just roughness without any chemical treatment. The surface geometry was determining factor in wetting contact angle analysis on electrospun meshes. We noted that it was very important how the geometry of electrospun surfaces was validated. The commonly used fiber diameter was not necessarily a convincing parameter unless it was correlated with the surface roughness or fraction of fibers or pores. Importantly, this study provides the guidelines to verify the surface free energy decrease with the fiber fraction for the meshes, to validate the changes in wetting contact angles. Eventually, the analysis suggested that meshes could maintain the entrapped air between fibers, decreasing surface free energies for polymers, which increased the contact angle for liquids with surface tension above the critical Wenzel level to maintain the Cassie-Baxter regime for hydrophobic surfaces.

## 1. Introduction

Repellency to water and other liquids, especially with lower surface tension, cannot be simply attributed only to the surface chemistry, therefore wetting is one of the most studied topics when considering surface topography. Wetting is defined by the contact angle measured for the liquid, where a liquid-vapor interface meets a solid surface and depends on surface roughness [1]. In nature, the hydrophobicity is often a combination of low surface energy surfaces and designed roughness [2,3]. Hierarchical roughness is observed in many plants with the hierarchically organized structure of surface [4], usually to increase natural abilities of water collection [5]. The air that is captured between nanostructured surfaces with designed roughness is minimizing the surface energy so wetting contact angles [6]. Electrospun three-dimensional (3D) meshes, characterized by large surface area to volume or mass ratio, have a range of applications where wetting and surface properties [7] are particularly important, such as filtration [8,9,10], membranes [11,12,13], structured composites [14,15], optical sensing [16], water [17,18,19], energy harvesting [20], drug delivery [21], and tissue engineering [22,23]. Wettability of tissue scaffolds plays an important role in the adsorption of proteins [24] and cell culture [25,26].

The typical approach in the production of superhydrophobic surfaces is increasing surface roughness, through chemical modifications [27] and tuning mechanical properties of materials, such as stiffness [28]. The first one, roughness, can be controlled on electrospun surfaces via decorating of fiber surfaces [29], wrinkling [30,31], introducing core-shell [32] or core-sheath structures [33], and various surface treatment methods [34,35], which brings us to the second case. The third option is stiffness that for polymer fibers depends on the orientation of crystallites along the fiber axis [36,37,38] and the degree of crystallinity [39], affecting wetting angles and liquid spreading [40,41].

Generally, surface free energy is correlated with surface deformation, which is sensitive to surface roughness and chemical composition [42]. The surface free energy of electrospun fibers can be controlled along with their main axis with the polymer chain alignment or their reorientation of functional groups in the polymer chains at the surface [37], which eventually changes their wetting [43,44] and adhesion forces [45] between them. The mesh is 3D structured network of fibers [35] with very high porosity, reaching often above 90% [14].

In this study, we investigated the wetting properties of the 3D meshes that were produced with randomly oriented electrospun polymer fibers. We present here the mechanistic study evaluating the wetting properties of meshes when considering the fiber diameter (*D*_f_) [46], roughness of meshes that is validated by arithmetical mean deviation (*R*_a_) [47], and a fraction of fibers in meshes (*F*_f_) [48], which is opposite of air volume fraction [49]. The fraction of fibers from two-dimensional (2D) images can give an estimate about the air captured between fibers at the surfaces in the meshes. Fibers morphology, *D*_f_ distribution, and *F*_f_ were analyzed based on scanning electron microscopy (SEM) micrographs. The aim of the study was to verify the commonly used fiber diameter of fiber to justify changes in the observed wetting angles. Instead of *D*_f_, we focused our analysis *R*_a_ and *F*_f_ giving characteristics of the larger contact area in contact with the liquid. Additionally, the surface free energy of polymer films and meshes was verified based on contact angle measurements using the Owens–Wendt theory and the surface chemistry of meshes and flat films was investigated with X-ray photoelectron microscopy (XPS).

Previously, many studies have shown the effect of increased contact angles, most often for water, for the decreased fiber diameter [7,46], however, the smallest fibers were often with beads and the variations of contact angles were smaller than 10° for the sizes from 0.6 to 2.2 µm [46] and between 3.5 to 8 µm another 10° [7]. Other studies actually showed the opposite, so an increase of contact angle with fibers diameter [50] or no correlation at all with *D*_f_ [51]. Based on the above-mentioned studies, we tried to identify the range of fiber diameter that should not influence wetting contact angle, so we could focus in our investigation on roughness and fraction of fibers effect on wetting of polymer meshes. The fraction of fibers controls the air capture on the surfaces of the mesh, which reduces the surface free energy and wetting angles. Herein, we found that *R*_a_ and *F*_f_ had a dominating effect on wetting of electrospun meshes, especially with low surface tension liquids. The *F*_f_ is directly correlated with the volume of air trapped at the surface of meshes, which defines the hydrophobicity for the Cassie-Baxter wetting regime [52,53] or hydrophlilicity for applicability of the Wenzel wetting theory [54]. In all of the wetting experiments on electrospun fibers, *D*_f_ should be correlated with the roughness parameters, and additionally *F*_f_ should be analyzed from the surface images. The *F*_f_ is directly correlated with the volume of air trapped at the surface of meshes, which is crucial to be considered for hydrophobic polymers in terms of the Cassie–Baxter wetting regime [52].

We show the importance of surface roughness analysis to interpret the contact angle data correctly, which are one of the most often experiments used in interfacial research. Notable, this study is highly desirable for several existing and emerging applications of porous meshes, as it identifies the advantages and limitations of fiber-based materials.

## 2. Materials and Methods

### 2.1. Polymer Solutions

The following seven polymers were used to produce films and fibers: poly(methyl methacrylate) (PMMA), poly(lactic-*co*-glycolic acid) (PLGA), polycarbonate (PC), polycaprolactone (PCL), polystyrene (PS), polyvinylidene fluoride (PVDF), and most hydrophilic nylon 6 (PA6). The polymer solutions were prepared on a magnetic stirrer with a heating plate (IKA RCT basic, Staufen, Germany) for electrospinning fibers and spin-coating films. Before preparing solutions, all of the polymers were dried at 70 °C for 24 h, except PCL at 45 °C and PS and PA6 at 40 °C. The parameters are stated in Table 1, with the following solvents used: formic acid, acetic acid, dimethylacetamide (DMAc), acetone, dimethylformamide (DMF), chloroform, and tetrahydrofuran (THF), all purchased from Sigma Aldrich, Gillingham, UK.

### 2.2. Electrospinning

Electrospinning of polymer fibers was carried out using apparatus EC–DIG with climate upgrade system (IME Technologies, the Netherlands) at constant temperature (*T*_c_) of 25 °C and relative humidity (*H*) between 35 and 60%, see Table 2 for further details. The applied voltage (*U*) to the nozzle, with an inner diameter of 0.8 mm, was in the range from 10 to 18 kV and the distance between the nozzle and the grounded collector (*d*) was from 10 to 20 cm. The flow rate for polymer solution (*Q*) was the lowest for PCL 0.001 ml·h^−1^, as previously indicated [55], and the highest for PLGA 9.5 ml·h^−1^.

The thickness of electrospun samples was controlled with the deposition time to obtain thickness from 14 to 600 μm, depending on initial *D*_f_. The substrate for deposition of fibers during electrospinning did not affect fibers’ morphology or size, therefore we used an aluminum foil for microscopy study, glass slides for roughness and contact angle measurements and Si wafers for chemical analysis. After electrospinning, the produced meshes were left for a few hours to dry in the air to ensure solvent residues evaporation in the case that any were left. All of the samples after preparation were stored in polystyrene Petri dishes that were placed in a desiccator.

### 2.3. Spin-Coating

To produce polymer films from the solutions (0.1–0.3 ml) listed in Table 1 we used spin-coated (L2001A v.3, Ossila, Sheffield, UK) set with rotation speed (*v*_s_) and spinning time (*t*_s_), as specified in Table 2. The samples were prepared on 16 × 16 mm glass slides for contact angle and on 15 × 15 mm Si wafers for surface chemistry analysis using XPS. The SEM micrographs of all spin-coated polymer films are in Figure S1 in the Supporting Information.

### 2.4. Surface Profilometry

Within the profilometry study, we verified the roughness and thickness of electrospun samples using laser microscopy (Olympus OLS4000, Tokyo, Japan), from a larger area [56] than usually is reached with AFM [57]. The measured area for all samples was 646 × 646 μm, except PA6, where it was 130 × 130 μm due to smaller fiber diameter. We obtained roughness average (*R*_a_), which is used to describe the roughness of measured surfaces, calculated with the digital approximation of:

(1) $$R_{a}=\frac{1}{\mathrm{MN}}\sum_{j=1}^{M}\sum_{i=1}^{N}\left|Z_{\mathrm{ij}}\right|$$

where *M* and *N* is a number of data points in X, Y direction, and Z is the surface height relative to the reference mean plate. The 2D images from profilometry analysis of all electrospun samples and thickness measurements are provided in the Supporting Information, Figure S2.

### 2.5. SEM Parameters and Image Analysis

**Figure 1:**
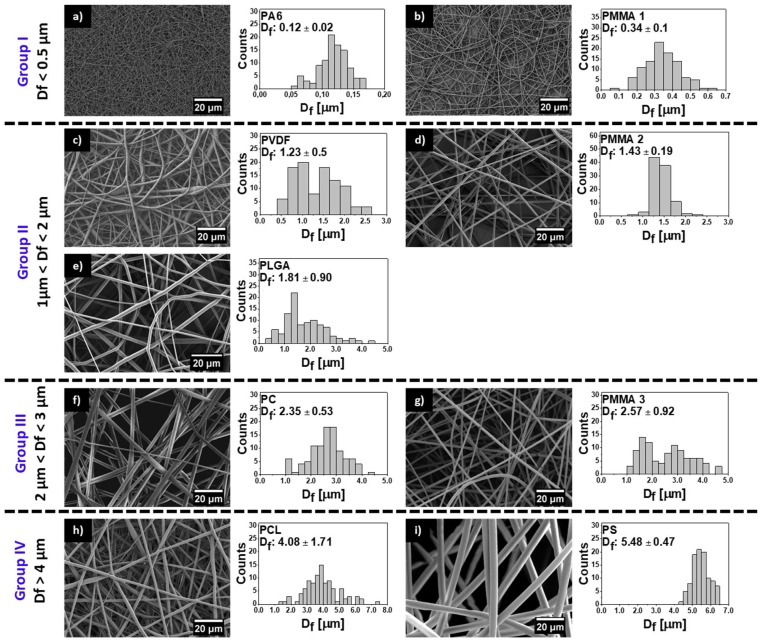
Scanning electron microscopy (SEM) images of electrospun fibers with fiber diameter (*D*_f_) histograms for (**a**) hydrophilic nylon 6 (PA6), (**b**) poly(methyl methacrylate) (PMMA) 1, (**c**) polyvinylidene fluoride (PVDF), (**d**) PMMA 2, (**e**) poly(lactic-*co*-glycolic acid) (PLGA), (**f**) polycarbonate (PC), (**g**) PMMA 3, (**h**) polycaprolactone (PCL), and (**i**) polystyrene (PS).

Prior to imaging with SEM, all samples of electrospun fibers and films were sputter coated with 5 nm gold layer using rotary-pump sputter coater (Q150RS, Quorum Technologies, Laughton, UK). We used an accelerating voltage of 3 kV and a current of 150 pA at a working distance of 6 mm in SEM (Merlin Gemini II, Zeiss, Germany). The average *D*_f_ was measured on 100 fibers using ImageJ [58] (v.1.51g) for all electrospun polymers with standard deviations, see histograms and micrographs in Figure 1 SEM micrographs of all spin-coated films are included in Supporting Information, see Figure S1. From SEM micrographs of electrospun surfaces, uploaded to ImageJ, we calculated *F*_f_. Prior, the images were made binary using DiameterJ plug-in for the best automatic threshold and next from the obtained images, pixels were counted using histogram plug-in. This ratio of white to black pixels indicated the *F*_f_. The representative binary images for *F*_f_ analysis are presented in Figure S3 in the Supporting Information.

### 2.6. Contact Angle and Surface Free Energy

Advancing contact angles on electrospun fibers and polymer films were measured by pipetting droplets of 3 μL volume on the surfaces using deionized (DI) water (Spring 5UV purification system—Hydrolab, Straszyn, Poland), glycerol (Pure, Sigma Aldrich), and formamide (Pure, Sigma Aldrich) for exact information, see Table 3. Experiments were carried out at a temperature of 21 °C and a humidity of 50 %. Immediately, after the liquid deposition, the images of droplets were taken using Canon EOS 700D camera with EF-S 60mm f/2.8 Macro USM zoom lens. The advancing contact angle was measured using ImageJ and MB Ruler (v5.3 MB-Software, Iffezheim, Germany) software. The representative images of contact angels for all samples and all liquids are shown in Table S1 in the Supporting Information.

The liquids: water, glycerol, and formamide were chosen for contact angle study because of their low vapor pressure and various polar and dispersive components of their surface tension, their surface tension components are shown in Table 3. The surface free energy was calculated using the Owens-Wendt approach [64], with the following equation [65,66]:

(2) $$\frac{\gamma_{l}\left(1+cos\theta \right)}{2\sqrt{\gamma_{l}^{d}}}=\sqrt{\gamma_{s}^{p}}\left(\frac{\sqrt{\gamma_{l}^{p}}}{\sqrt{\gamma_{l}^{d}}}\right)+\sqrt{\gamma_{s}^{d}}$$

where $\gamma_{l}$ is the total surface tension of the probe liquid with dispersive $\gamma_{l}^{d}$ and polar $\gamma_{l}^{p}$ components and $\gamma_{s}$ is the surface free energy of a solid surface with dispersive $\gamma_{s}^{d}$ and polar $\gamma_{s}^{p}$ components. *Ɵ* is the contact angle between the probe liquid and solid surface. The error in the contact angle data was calculated based on standard deviation, which was later used for the estimation of surface free energy error, which was approximately 5.6%.

### 2.7. X-ray Photoelectron Spectroscopy

The XPS analyses were carried out in a PHI VersaProbe II Scanning XPS system using monochromatic Al Kα (1486.6 eV) X-rays focused to a 100 µm spot and scanned over the sample area of 400 × 400 µm. The photoelectron take-off angle was 45° and the pass energy in the analyzer was set to 23.50 eV to obtain high energy resolution spectra for the C 1s, O 1s, N 1s, and F 1s regions. A dual beam charge compensation with 7 eV Ar^+^ ions and 1 eV electrons were used to maintain a constant sample surface potential, regardless of the sample conductivity. All XPS spectra were charge referenced to the unfunctionalized, saturated carbon (C–C) C 1s peak at 284.8 eV. The operating pressure in the analytical chamber was less than 4x10^−9^ mbar. Deconvolution of spectra was carried out using PHI MultiPak software (v.9.7.0.1). Spectrum background was subtracted using the Shirley method.

### 2.8. Experimental Summary

In order to characterize the wetting properties of electrospun meshes, we performed a number of mechanistic experiments, including:

1. Investigation of fibers and films morphology using SEM; fiber diameter comparison to surface roughness and fiber fraction for all meshes.
2. Wetting contact angle on films and meshes made of seven types of polymer (including PMMA, PLGA, PC, PCL, PS, PVDF, and most hydrophilic PA6), without any surface modifications; meshes were divided to four groups by their average fiber diameter below 0.5 µm, 1–2 µm, 2–3 µm, and 4–6 µm.
3. The varying fiber diameter of the same polymer (PMMA) for the three groups: below 0.5 µm, between 1–2 µm, and 2–3 µm, and keeping the same thickness of the meshes to validate wetting contact angle.
4. Analyzing the effect of thickness of 3D meshes on wetting contact angles.
5. Calculations of surface free energy and its changes with fiber fraction in the meshes, with additional surface chemistry analysis using XPS.

## 3. Results

### 3.1. Contact Angle versus Fiber Diameter, Roughness and Fiber Fraction

**Figure 2:**
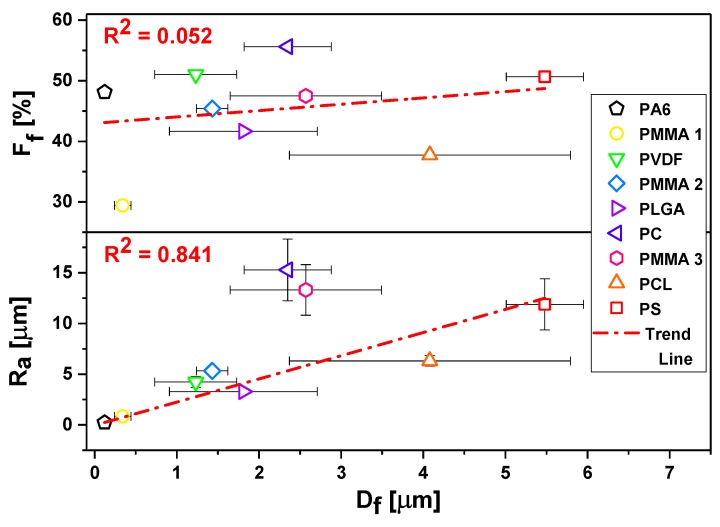
Roughness *R*_a_ and fraction *F*_f_ as a function of average fiber diameter, *D*_f_, in three-dimensional (3D) meshes.

The surface morphology of all meshes including hydrophobic PMMA, PLGA, PC, PCL, PS, PVDF, and hydrophilic PA6 are shown in Figure 1. We investigated the wetting of these meshes to verify the effect of surface chemistry without any additional surface modifications on the contact angles. All of the electrospun samples were divided into four groups in relation to obtained average *D*_f_: Group I with *D*_f_ below 0.5 μm, Group II with *D*_f_ between 1 and 2 μm, Group III with *D*_f_ between 2 and 3 μm, and Group IV with *D*_f_ between 4 and 6 μm, as shown in Figure 1, together with SEM images. The smallest average fiber diameters were obtained for PA6 and the largest for PS with *D*_f_ of 0.12 ± 0.2 μm and 5.48 ± 0.47 μm, respectively. The mean diameters for the rest of the electrospun fibers were as follows 0.34 ± 0.09 μm for PMMA 1, 1.23 ± 0.50 μm as shown before [67], for PVDF, 1.43 ± 0.19 μm, which confirmed our previous results [68], a for PLGA, 2.35 ± 0.53 μm for PC, 2.57 ± 0.92 μm for PMMA 3, and 4.08 ± 1.71 μm for PCL, see histograms in Figure 1. The average fiber diameter for PCL was 4.08 ± 1.71 μm, and for PS was 5.48 ± 0.47 μm. Notable, all electrospun fibers were without beads. As *D*_f_ is commonly used in wetting studies of electrospun surfaces we correlated it with roughness *R*_a_ and fiber fraction, *F*_f_, see Figures S2 and S3 in Supporting Information. To analyses *D*_f_ influence on sample roughness, we plotted *R*_a_ and *F*_f_ as a function of *D*_f_, see Figure 2. *R*_a_ was proportional to *D*_f_, however, there was almost no correlation *F*_f_ to *D*_f_.

**Figure 3:**
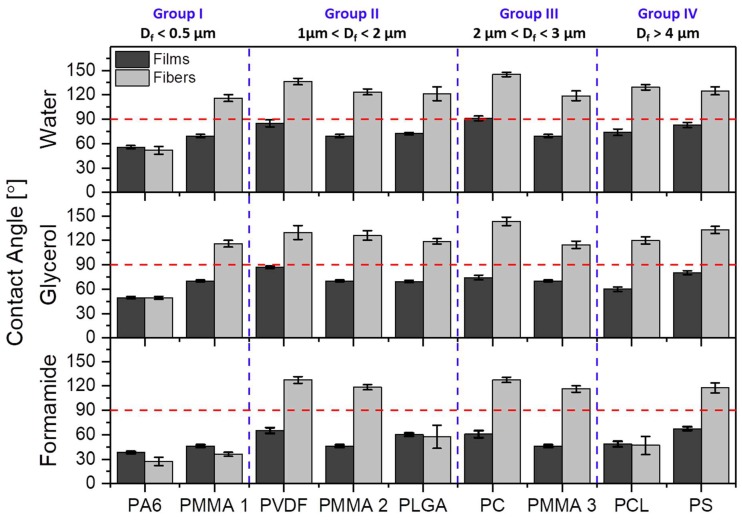
Contact angles values on polymer films and meshes for water, glycerol, and formamide. The horizontal red dashed lines indicate the threshold contact angle of 90° between hydrophobic and hydrophilic properties and perpendicular blue dashed lines indicate the four groups according to the average fiber diameter, *D*_f_.

The obtained contact angles on films and randomly oriented electrospun meshes were compared for all polymers in histogram that is presented in Figure 3. The SEM images, showing the smooth surfaces of polymer films, are presented in Figure S1 in the Supporting Information. The contact angles on films were close to hydrophobic for PVDF, PC, and PS, the rest of the investigated polymers exhibited contact angles that were much lower than 90°. We observed a straightforward increased of contact angles with all liquids on electrospun fibers when compared to films made out of the same polymer solution, except PA6 with formamide. PA6 fibers exhibited lower contact angle values when compared to films, because we observed the liquids percolation into fiber networks, similarly it happened for PMMA 1 mesh (all in Group I) for which formamide penetrated the membrane completely. For the PLGA in Group II and PCL in Group IV, we observed significantly lower contact angles being 57.4 ± 14.2° and 46.8 ± 11.2°, respectively, than for other electrospun fibers with similar *D*_f_. For all three liquids, the highest contact angles were observed for PC, within Group III, with values of 145.1 ± 2.6°, 143.3 ± 5.2°, and 127.3 ± 3.3° for water, glycerol, and formamide, respectively.

**Figure 4:**
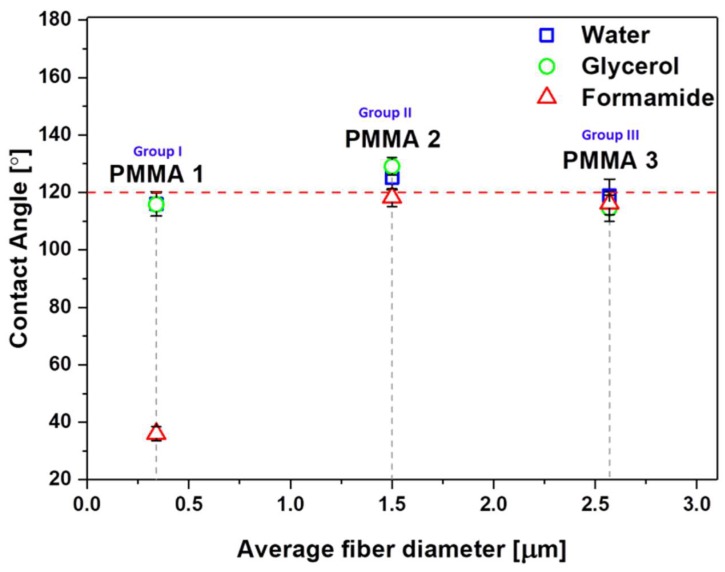
The contact angle for water, glycerol, and formamide on PMMA mesh with three sets of *D*_f_: PMMA 1—0.34 μm, PMMA 2—1.43 μm, PMMA 3—2.57 μm.

As the correlation between *D*_f_ and wetting contact angle might be affected by polymers variations, we verified it for PMMA only. For PMMA meshes, with three characteristic *D*_f_ from the first three groups (I-III), we obtained similar contact angles, close to 120° (Figure 4), with water and glycerol, having different *D*_f_, (Figure 1b,d,g), like in the previous study [51]. In the case of formamide with the surface tension of 58 mJm^−2^ with the lowest polar component among all of the liquids that we used for testing (see Table 3), the contact angle drastically decreases, and the liquid percolated into the network of fibers. It happened also with hexadecane (surface tension = 27.8 mJm^−^^2^) for the electrospun poly(2,2,2-trifluoroethyl methacrylate) (PTFEMA) with *D*_f_ of 500 nm [69], where liquid soaked into PTFEMA network.

Notable, the contact angles were not affected by the thickness of the electrospun samples. The PMMA 2 fibers with the same fiber diameter were deposited between 15 and 45 min to produce thickness between 35 and 134 μm of electrospun mats, see Table 4. By varying the deposition time of electrospinning, we slightly increased the PMMA 2 fiber diameter, thus *R*_a_ increased too, but *F*_f_ was in the same range. The SEM micrograph and *D*_f_ histograms of PMMA fibers with thickness variations are included in Figure S4, in the Supporting Information. The obtained contact angles with water, glycerol, and formamide were very similar for all PMMA samples, regardless of the thickness of the mesh, see Table 4.

**Figure 5:**
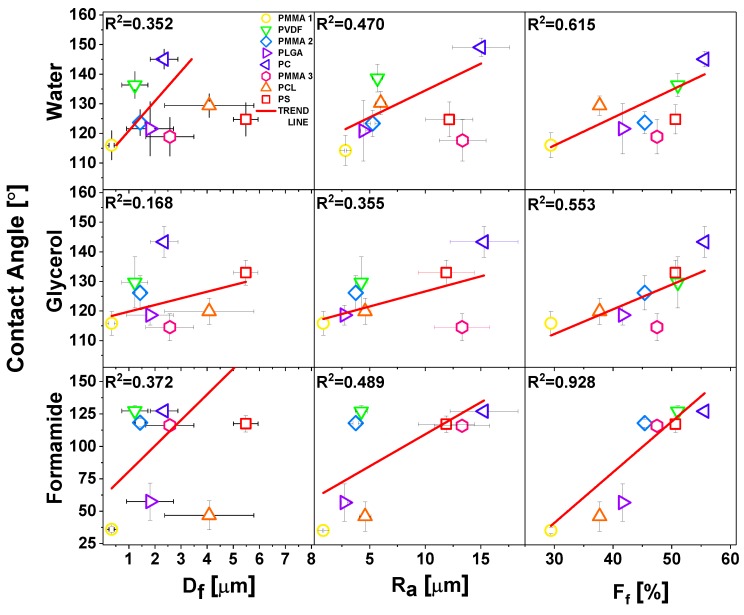
Water, glycerol, and formamide contact angles as a function of fiber diameters, *D*_f_ roughness *R*_a_, and *F*_f_. The linear fittings to the experimental points with *R*^2^ values are indicated on the graphs.

Regardless of the polymer type, the decrease of fiber diameter did not cause the increase of the water contact angle, as it was suggested in other studies [69]. Noteworthy is a fact that all of the fibers we investigated in this study were beadless. Beads on fibers usually occur in unstable electrospinning process [70], having often the size of few microns, which significantly changes *R*_a_ and *F*_f_, therefore the wetting properties of meshes. In Figure 5, we compared the effect of roughness parameters and contact angles for water, glycerol, and formamide for hydrophobic polymers, thus omitting hydrophilic PA6, as there was no significant difference in wetting between films and fibers. The less direct correlation between the contact angles was observed for *D*_f_, but there was a relatively good correlation between *R*_a_ and *F*_f._ However, *D*_f_ is correlated with *R*_a_, see Figure 3, and, with increased *D*_f_, *R*_a_ was proportionally increased. In all of the wetting experiments on electrospun fibers, *D*_f_ should be correlated with the roughness parameters, and additionally, *F*_f_ should be analyzed from the surface images. The *F*_f_ is directly correlated with the volume of air that is trapped at the surface of meshes, which is crucial to be considered for hydrophobic polymers in terms of the Cassie-Baxter wetting regime [52].

Despite the trend of using *D*_f_ as an indication of variations in the wetting of electrospun fibers, we identified *R*_a_ and *F*_f_ as the key parameters in the analysis of contact angles on electrospun meshes. Most of the previous studies on wetting of electrospun fibers were performed with water, therefore in Table 5, we summarized the literature data as compared with our results for this liquid. For the hydrophilic PA6, we obtained very similar contact angle of 50° with a diameter of 12–15 nm [71]. PVDF membrane with an average fiber diameter of 0.17 µm gave a contact angle of 142.8° [72], which was in the same range to what we measured, but on the PVDF fibers with diameter one order of magnitude higher: 1.23 µm. Similarly, PCL fibers had vastly lower fiber diameter, but the water contact angle was similar [73]. The wetting investigation of PLGA fibers yielded similar results [74]. In the case of PS, we obtained the contact angles of 124.8° with a diameter that was half as small as Kang et al. [75], having 12.7 µm and the contact angle of 138.1°. Regarding PC fibers [76], almost 20° difference in contact angle was observed for fibers with diameter of 1.6 µm smaller than what we produced. Additionally, in Figure 3, we showed the results for all electrospun PMMA samples that were actually very similar to previously obtained results for water [51,77] (see Table 5), confirming the same trend of contact angle independence from *D*_f_. The contact angle for PLGA, PCL, and PVDF fibers, as reported in Table 5, were almost identical for different *D*_f_.

### 3.2. The Surface Free Energy of Polymer Films

**Figure 6:**
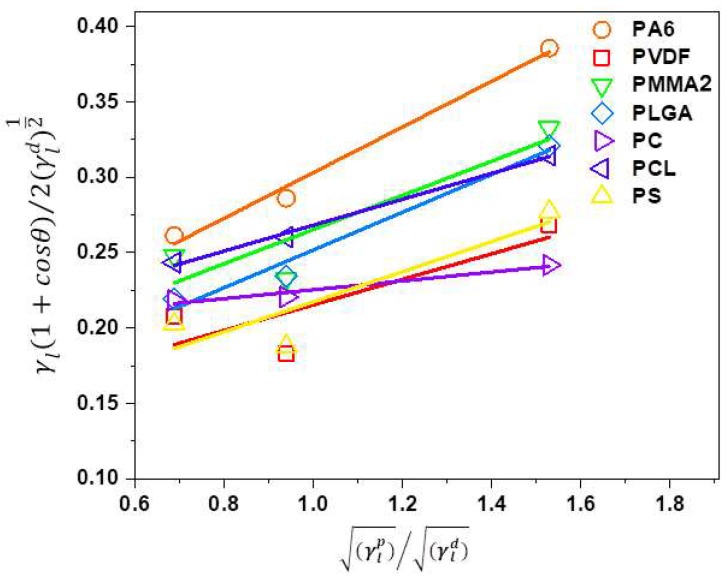
Owens–Wendt plots for all polymer films produced using Equation 2 for three probe liquids of different surface tension. The fitting function for the trend lines is presented in Supporting Information in Table S2.

Surface free energy is often calculated based on contact angle measurement using several liquids with different surface tension [78,79]. Notably, the surface free energy depends on the polymer chains organization that is related to the manufacturing methods used to produce films and fibers [43,44]. In this study, the obtained contact angle data on polymer films with water, glycerol, and formamide were used to calculate surface free energy based on the Owens–Wendt [64] method with polar and dispersive contributions. In Figure 6, the Owens-Wendt plots are presented and all of the fitting parameters are included in the Supporting Information in Table S2. The summary of all the calculated polar and dispersive components of surface free energies of all investigated polymer films are shown in Table 6. Additionally, Table 6 includes the values found in the literature for given polymers.

We obtained for PA6 films a very similar surface free energy of 45.9 mJm^−2^ as compared to 40.3 mJm^−2^ [44]. The lowest surface free energy of 24.2 mJm^−2^ was for PVDF in comparison to slightly higher values that were obtained by Wu [80], who used only two liquids to measure the contact angles: water and diiodomethane and calculated surface free energy. Concerning PMMA film, Wu [81] used molten polymer and obtained surface free energy of 32 mJm^−^^2^, which was comparable to our results, see Table 6. Surface free energy for PC was simulated using contact angle hysteresis model giving the result of 44.0 mJm^−2^ similar to our 39.3 mJm^−2^ [27]. Luk et al. [82] used water, ethylene glycol, and DMSO to measure contact angles on PCL and obtained surface free energy of 33 mJm^−^^2^, which was confirmed by slightly higher values of 40.7 mJm^−2^, which are indicated in Table 6. The PS films were characterized with the surface free energy of 25.3 mJm^−2^, which were lower than the 32.1 mJm^−2^ obtained from measurements at a temperature of 140 °C [81]. Often, the differences in polar and dispersive part of surface free energy were associated with a diversity of liquid surface tensions used for contact angle measurement and calculation methods. The surface free energy data varied and was strongly dependent on calculations methods and liquids that were used for contact angle measurements [78].

The surface free energy of electrospun fibers may slightly differ from films, as was proven for PA6 fibers, showing increased polar components [44,45]. The lowest polar component of surface free energy suggested that most of the liquid interactions with electrospun fibers can be related to the dispersive part including van der Waals forces, enhanced by the large surface area created by the small diameter of fibers. We observed this effect for PA6 and PMMA fibers, from Group I, see Figure 3. A similar effect was partially observed by Cho et. Al., who studied the porosity of electrospun mats effect on contact angle using a mixture of water and ethanol. The controlled addition of ethanol decreased the surface tension of mixture and thus also decreased of contact angle on electrospun samples [83]. For PCL the critical surface tension of liquids was 57 mNm^−1^ [7], which is in the range of formamide, see Table 3, thus the contact angles with formamide on PCL meshes are much lower, similarly for PLGA. The critical surface tension is related to the Wenzel state transition from the Cassie–Baxter regime when liquids start to penetrate the network of fibers [53,54].

**Figure 7:**
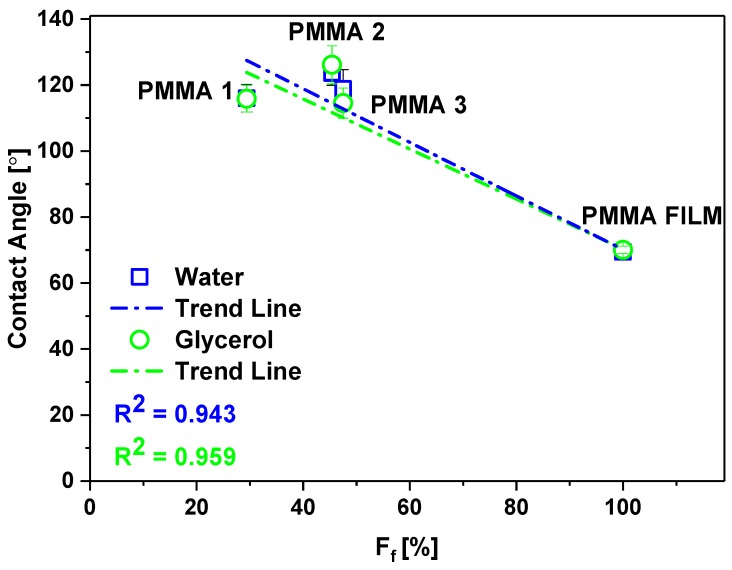
Changes in contact angle for water and glycerol on PMMA samples with a fraction of fibers in 3D meshes.

Keeping in mind that we investigated here high porosity meshes built of a 3D network of randomly arranged fibers we analyzed the fraction of fibers in contact with liquid droplets in wetting experiments. When considering electrospun PMMA meshes as hydrophobic surfaces (Figure 3 and Figure 4), with various fiber fraction, (Figure 5) we analyzed the samples based on Cassie–Baxter state [53]. In Figure 7, we plotted the wetting contact angles with water and glycerol only for PMMA samples characterized by a different fraction of fiber, for PMMA film, we assume *F*_f_ equal 100%. As a result, the wetting contact angles decreased proportionally to the decreased *F*_f_. Therefore, we propose a statement that surface free energies decrease with the increased air fraction trapped between fibers building the 3D meshes, and the wetting contact angles are increasing.

Our analysis of surface irregularities show that providing only *D*_f_ values of electrospun fibers for wetting analysis is not sufficient and additional parameters such as *F*_f_ or *R*_a_ should be considered to understand the wetting properties of 3D meshes. Surface free energy is reduced here by the air that can be clearly analyzed with the *F*_f_ for meshes, as shown in Figure 7. Importantly, the decreased *F*_f_ gives lower values of surface free energy and the increase of contact angles. We showed the direct correlation that can be further exploited in terms of theoretical descriptions to provide the prediction of the wetting behavior of meshes with designed fiber fractions.

### 3.3. Surface Chemistry

The surface chemistry of electrospun polymer fibers and films were analyzed using the XPS method. Atomic concentrations of constituents at the surface of all studied polymer fibers and their film equivalents are presented in Table 7. Results for polymer fibers show slightly increased oxygen content at the surface as compared with the films, except for PCL, for which the oxygen/carbon (O/C in Table 7) ratio was insignificantly decreased. Additionally, for all studied polymers excluding PCL, the fiber compositions were closer to theoretically expected values. However, it was unfeasible to indicate any clear dependence between the surface chemistry of fibers and films, their oxygen, fluorine, and/or nitrogen content changed, in relation to measured contact angles. The change in the surface compositions among films and fibers had a negligible impact on measured contact angles.

## 4. Conclusions

The aim of this study was to validate surface geometry effects over the polymer chemistry on contact angles on electrospun meshes. We found that it was very important how the geometry of electrospun surfaces was validated. The commonly used fiber diameter was not necessarily a convincing parameter unless it was correlated with surface roughness [7,46,50,51,69]. Another important parameter that should be included in the roughness analysis of electrospun surfaces was a fraction of fibers that was strictly correlated with changes of surface free energies of 3D meshes, which was reduced by the air trapped between fibers.

We showed that the wetting of electrospun meshes was eventually controlled with roughness and fraction of fibers, which was related to the surfaces in contact with liquids. The air trapped between fibers was keeping their wetting behavior in the Cassie–Baxter regime, however, it was quickly transformed to the Wenzel state once the liquid surface tension was reduced to 58 mNm^−1^. Increasing roughness of the most hydrophilic polymers, represented here by PA6, was not sufficient to achieve higher contact angles than on films, suggesting that chemical modifications would be necessary to obtain higher contact angles on PA6 meshes. In the case of formamide, where surface tension was close to the critical values for given polymer, the dispersive and polar components played an important role for liquids and solids. The van der Waals interaction included in the dispersive part of surface free energy was enhanced due to the increased surface area of electrospun samples, which resulted in liquids soaking into the small diameter (below 0.5 µm) fiber networks. The hydrophobic properties of the surfaces can be enhanced by roughness that is created by randomly oriented fibers without complex chemical treatment. The geometry was the main factor influencing the contact angle of high surface tension liquids, such as water and glycerol on electrospun fibers, unaffected by polymer surface chemistry.

Our study opens the discussion about the standard methods to use in the characterization roughness of electrospun fibers roughness to be able to compare it to wetting contact angles. Within this comprehensive study, we indicated the complexity of surface effects on wetting on electrospun fibers from polymers that are very often used in the filtration and biomedical industry.

## Figures and Tables

**Table 1:** Details of solution preparation for electrospinning and spin-coating for all polymers including molecular weight (*M*_w_), concentration *w*/*w* (C), solvents, stirring time (*t*), the rotation speed of stirrer (*v*_r_), and temperature of the hot plate during mixing.

Polymer	Supplier	*M*_w_ [g·mol^−1^]	Solvents	*C* [%]	*t* [h]	*v_r_* [rpm]	*T* [°C]
PA6	BASF, Ludwigshafen, Germany	24,000	Formic Acid:Acetic Acid 1:1	12	4	500	25
PVDF	Sigma Aldrich	275,000	DMAc:Acetone 1:1	22	4	1500	60
PMMA 1	Sigma Aldrich	150,000	Formic Acid:(DMF) 7:3	12	3	750	45
PMMA 2	Sigma Aldrich	350,000	DMF	12	2.5	500	55
PMMA 3	Sigma Aldrich	150,000	DMF	30	3	750	45
PLGA	Sigma Aldrich	66,000–107,000	Chloroform:DMF 85:15	15	2	500	25
PC	Goodfellow, Huntingdon, UK	-	DMF:THF 1:1	20	4	900	50
PCL	Perstorp, Warrington, UK	50,000	Chloroform	12	2	700	25
PS	Sigma Aldrich	350,000	DMF	25	6	500	25

**Table 2:** The electrospinning and spin-coating parameters used to produce polymer fibers and films.

Polymer	Electrospinning				Spin-Coating	
	*U* [kV]	*Q* [ml·h^−1^]	*D* [cm]	*H* [%]	*t*_s_ [s]	*v*_s_ [rpm]
PA6	16	0.2	15	40	10	1000
PVDF	15	4.2	18	60	40	3500
PMMA 1	11	0.3	10	40	-	-
PMMA 2	12	4.0	15	35	20	3500
PMMA 3	12	3	15	40	-	-
PLGA	17	9.5	15	60	60	3000
PC	12	3.0	15	50	20	5000
PCL	14	0.001	20	50	60	3000
PS	11	1.5	15	40	10	2000

**Table 3:** Surface tension and its components for all liquids: water [59,60], glycerol [61,62], and formamide [62,63] used in contact angle measurements.

Liquid	$\gamma_{l}$ [mJm^−2^]	$\gamma_{l}^{p}$ [mJm^−2^]	$\gamma_{l}^{d}$ [mJm^−2^]
**Water**	72.8	51.0	21.8
**Glycerol**	64.0	30.0	34.0
**Formamide**	58.2	18.7	39.5

**Table 4:** The thickness of electrospun samples from PMMA 2 fibers with characteristic electrospinning time and all other characteristic parameters, such as *D*_f_, *R*_a_, *F*_f_, and contact angles with water, glycerol, and formamide.

Fibers Deposition Time [min]	Sample Thickness [μm]	*D*_f_ [μm]	*R*_a_ [µm]	*F*_f_ [%]	Contact Angle [°]		
					Water	Glycerol	Formamide
15	35.32	1.43 ± 0.25	7.10 ± 0.91	45.20 ± 15.55	131.9 ± 3.3	129.1 ± 3.0	118.2 ± 3.1
30	67.90	1.55 ± 0.25	6.76 ± 0.52	45.41 ± 3.14	125.2 ± 4.2	126.1 ± 5.8	119.6 ± 2.3
45	134.68	1.70 ± 0.20	10.18 ± 0.82	41.18 ± 10.75	130.3 ± 5.0	126.7 ± 2.8	118.6 ± 3.1

**Table 5:** Water contact angle on electrospun polymer fibers with *D*_f_ as compared with the literature data.

Polymer Fibers	Water Contact Angle [°]		D_f_ [μm]	
	Measured	Literature Data with References	Measured	Literature Data with References
PA6	51.7 ± 4.9	50.0 [71]	0.12 ± 0.02	0.15 ± 0.03 [71]
PVDF	136.3 ± 3.9	142.8 ± 1.4 [72]	1.23 ± 0.50	~0.17 [72]
PMMA 1	116.0 ± 4.2	134.6 ± 3.0 [51]	0.34 ± 0.09	0.34 ± 0.05 [51]
PMMA 2	125.2 ± 4.2	132.2 ± 4.1 [51]	1.43 ± 0.19	1.42 ± 0.14 [51]
PMMA 3	118.8 ± 5.8	135.0 [77]	2.57 ± 0.92	5.00 - 5.50 [77]
PLGA	121.6 ± 8.5	125.0 [74]	1.81 ± 0.90	0.80 - 1.60 [74]
PC	145.1 ± 2.6	122.0 ± 0.7 [76]	2.35 ± 0.53	~0.75 [76]
PCL	129.4 ± 3.3	128.0 ± 6.0 [73]	4.08 ± 1.71	1.30 ± 0.60 [73]
PS	124.8 ± 4.9	138.1 ± 0.7 [75]	5.48 ± 0.47	12.70 ± 1.60 [75]

**Table 6:** Summaries of surface free energy calculated for all polymer films based on Owens-Wendt plots shown in Figure 6 with data found in the literature and references.

Polymer	Surface Free Energy [mJm^−2^]						
	Calculated			Literature Data			
	$\gamma_{s}$	$\gamma_{s}^{p}$	$\gamma_{s}^{d}$	$\gamma_{s}$	$\gamma_{s}^{p}$	$\gamma_{s}^{d}$	Ref.
PA6	45.9	22.9	23.0	40.3	5.50	34.9	[44]
PVDF	24.2	7.20	17.1	30.3	7.00	23.3	[80]
PMMA	35.9	12.8	23.1	32.0	9.00	23.0	[81]
PLGA	31.7	15.7	16.0	-	-	-	-
PC	39.3	0.90	38.5	44.0	1.00	43.0	[27]
PCL	40.7	7.40	33.3	33.0	10.0	22.0	[82]
PS	25.3	9.10	16.2	32.1	5.40	26.7	[81]

**Table 7:** X-ray photoelectron microscopy (XPS) results, expressed as % atomic, for polymer films and fibers prepared from the same polymer solution for PA6, PVDF, PMMA, PLGA, PC, PCL, and PS.

Polymer Sample		C	O	F	N	O/C
PA6	fibers	75.2	11.9	-	12.9	0.16
	film	78.0	10.2	-	11.8	0.13
PVDF	fibers	51.1	0.70	48.1	-	-
	film	53.2	-	46.8	-	-
PMMA 2	fibers	69.5	29.9	-	-	0.43
	film	73.4	26.6	-	-	0.36
PLGA	fibers	60.1	39.9	-	-	0.66
	film	61.6	38.4	-	-	0.62
PC	fibers	82.9	17.1	-	-	0.21
	film	85.2	14.8	-	-	0.17
PCL	fibers	78.0	22.0	-	-	0.28
	film	77.5	22.5	-	-	0.29
PS	fibers	100.0	0.00	-	-	0.00
	film	100.0	0.00	-	-	0.00

## Supplementary Materials

The following are available online at http://www.mdpi.com/2073-4360/11/1/34/s1, Figure S1: SEM micrographs of spin-coated polymer films: (a) PA6, (b) PVDF, (c) PMMA, (d) PLGA, (e) PC, (f) PCL, and (g) PS; Figure S2: Surface images from laser microscope for (a) PA6, (b) PVDF, (c) PMMA 1, (d) PMMA 2, (e) PMMA 3, (f) PLGA, (g) PC, (h) PCL, (i) PS used for roughness measurements Ra, Figure S3: Binary images of electrospun meshes obtained from SEM images that were used for the calculation of fiber fraction, Ff for (a) PA6, (b) PVDF, (c) PMMA 1, (d) PMMA 2, (e) PMMA 3, (f) PLGA, (g) PC, (h) PCL, (i) PS; Figure S4: Electrospun PMMA fibers deposited for (a) 15 minutes, (b) 30 minutes, (c) 45 minutes with fiber diameters’ histograms, Table S1: Representative images of a droplet on electrospun polymer fibers and films with water, glycerol, and formamide used for contact angle measurements with the average values of contact angle ± standard deviation for all the samples, Table S2: A linear fit to Owens–Wendt plots presented in Figure 4. The intercept and slope were used for the calculation of polar and dispersive components of surface free energy (SFE).
