# Supplementary material for: Roughness and Fiber Fraction Dominated Wetting of Electrospun Fiber-Based Porous Meshes
Source: Polymers (Basel). 2018 Dec 27;11(1):34. doi: 10.3390/polym11010034 (PMC6401689; doi:10.3390/polym11010034)
Supplement: Supplementary file 1 [file polymers-11-00034-s001.pdf]

Supporting Information to

Roughness and fiber fraction dominated wetting of electrospun fiber based porous meshes

Piotr K. Szewczyk[1], Daniel P. Ura[1], Sara Metwally[1], Joanna Knapczyk-Korczak[1], Marcin Gajek[2], Mateusz M. Marzec[3], Andrzej Bernasik[3,4], Urszula Stachewicz[1]*

[1] International Centre of Electron Microscopy for Materials Science, Faculty of Metals Engineering and Industrial Computer Science, AGH University of Science and Technology

[2] Faculty of Materials Science and Ceramics, AGH University of Science and Technology

[3] Academic Centre for Materials and Nanotechnology, AGH University of Science and Technology, Poland

[4] Faculty of Physics and Applied Computer Science, AGH University of Science and Technology, Poland

* Corresponding author email: ustachew@agh.edu.pl; Tel.: + 48 12 617 5230

Keywords: roughness; contact angle; electrospinning; fiber; fraction; surface free energy; wetting

In this supporting data we provided SEM micrographs of smooth polymer films used for contact angle measurement (Figure S1), original images from laser microscope used for R_a calculations (Figure S2) along with the binary images used for fraction of fibers calculations (Figure S3), and SEM images with respective histograms of different thickness of PMMA meshes (Figure 4S). In (Table 1S) representative images of measured contact angles are presented and in (Table 2S) the original linear fit to data used for surface free energy calculations are listed.

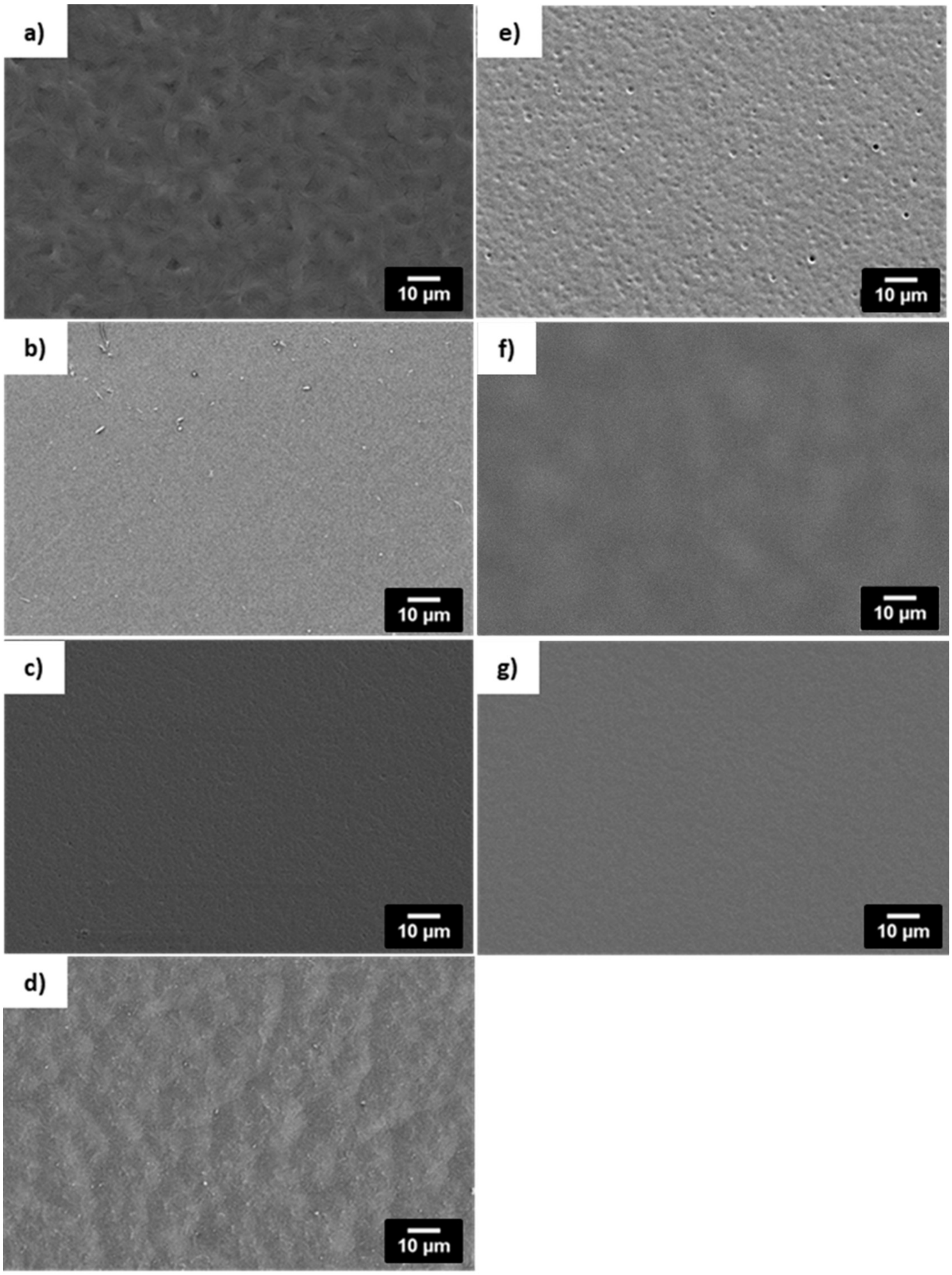

Figure S1. SEM micrographs of spin-coated polymer films: a) PA6, b) PVDF, c) PMMA, d) PLGA, e) PC, f) PCL, and g) PS.

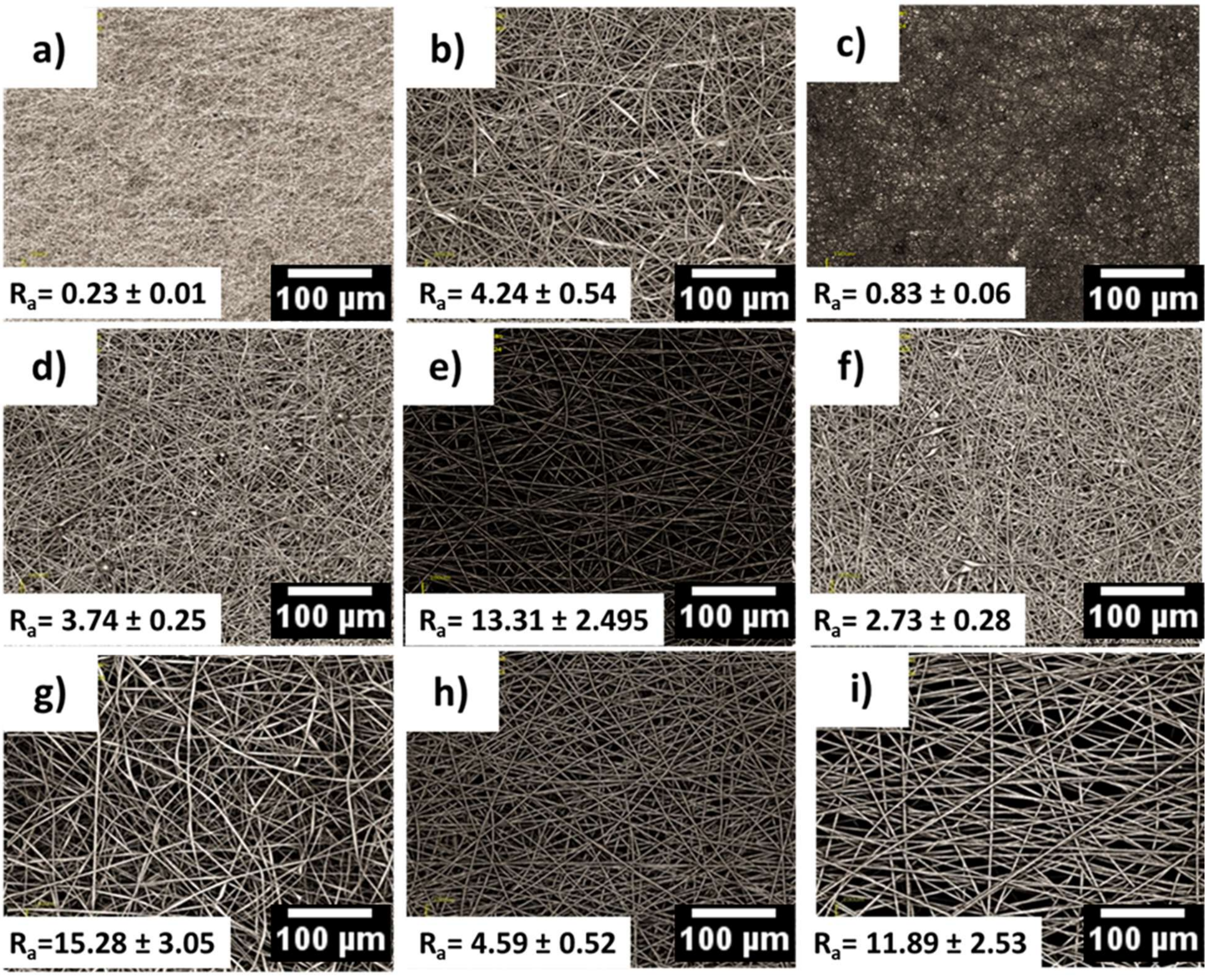

Figure S2. Surface images from laser microscope for a) PA6, b) PVDF, c) PMMA 1, d) PMMA 2, e) PMMA 3, f) PLGA, g) PC, h) PCL, i) PS used for roughness measurements R_a.

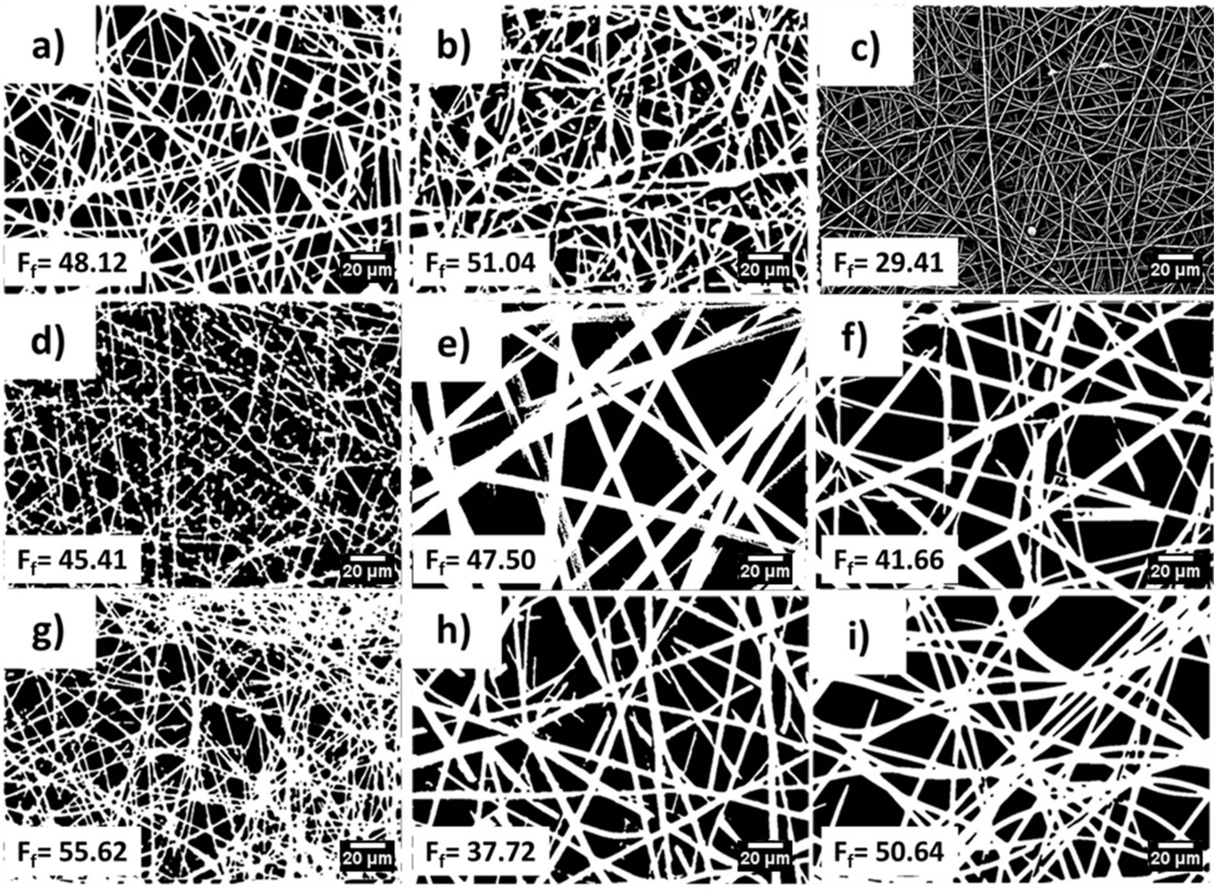

Figure S3. Binary images of electrospun meshes obtained from SEM images that were used for the calculation of fiber fraction, F_f for a) PA6, b) PVDF, c) PMMA 1, d) PMMA 2, e) PMMA 3, f) PLGA, g) PC, h) PCL, i) PS.

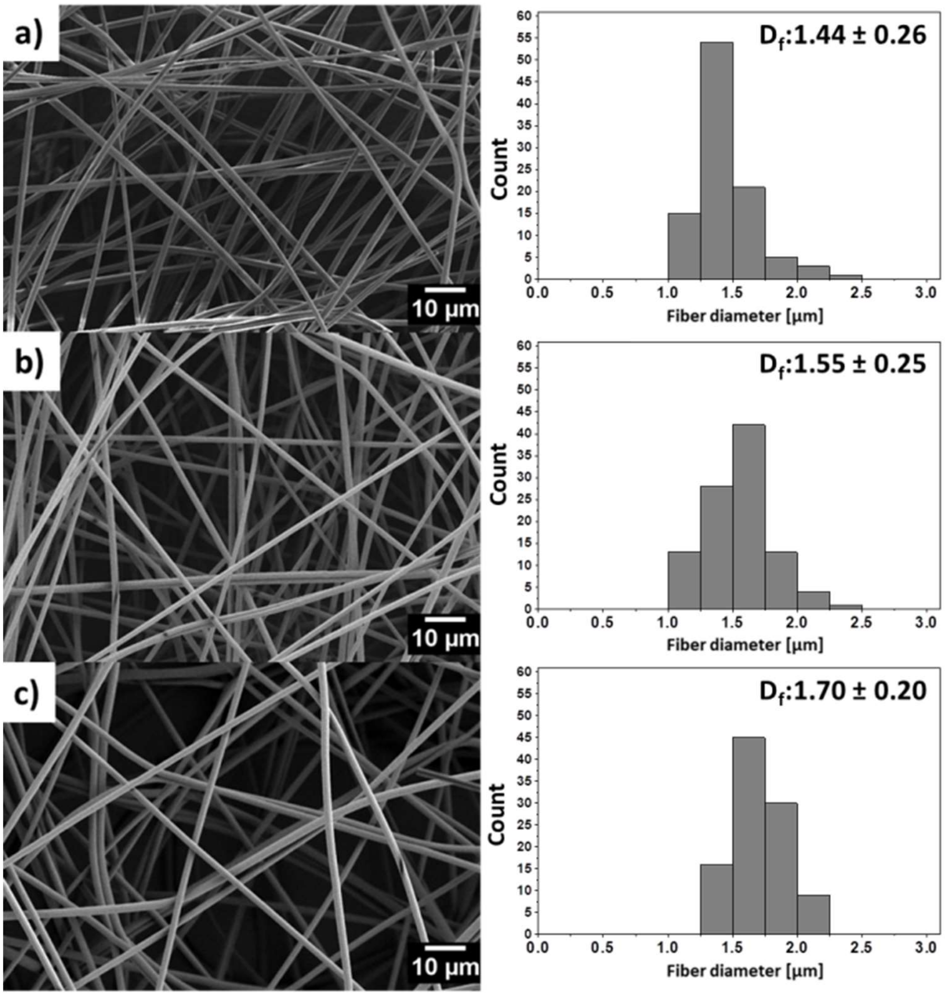

Figure S4. Electrospun PMMA fibers deposited for a) 15 minutes, b) 30 minutes, c) 45 minutes with fiber diameters' histograms.

Table S1. Representative images of a droplet on electrospun polymer fibers and films with water, glycerol, and formamide used for contact angle measurements with the average values of contact angle ± standard deviation for all the samples.

Polymer Sample			Contact Angle [°]		
			Water	Glycerol	Formamide
PA6	Fibers		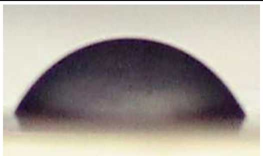 51.65 ± 4.86	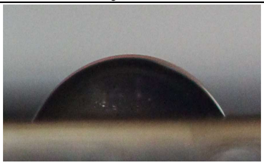 49.44 ± 1.80	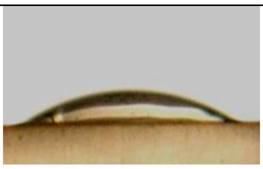 27.32 ± 5.23
	Films		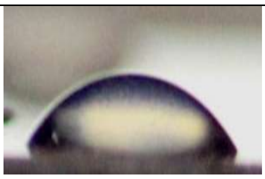 55.63 ± 1.79	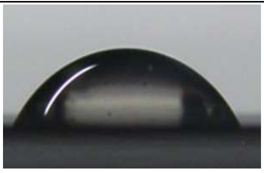 49.61 ± 1.50	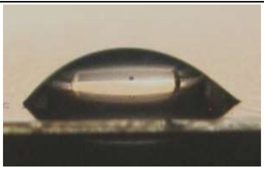 38.25 ± 1.14
PVDF	Fibers		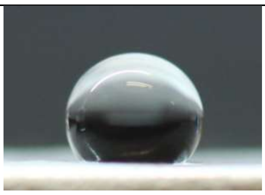 136.34 ± 3.85	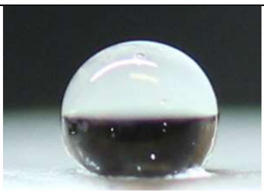 129.67 ± 8.62	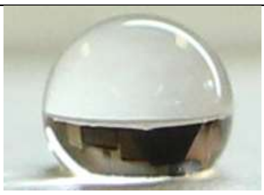 127.27 ± 4.19
	Films		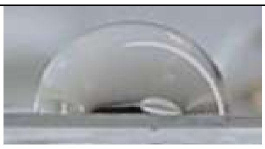 84.95 ± 4.43	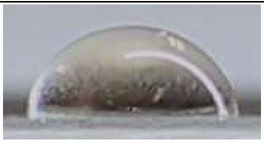 86.83 ± 1.67	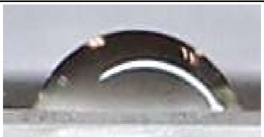 65.16 ± 3.55
PMMA	PMMA 1 Fibers		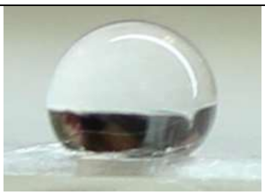 116.02 ± 4.20	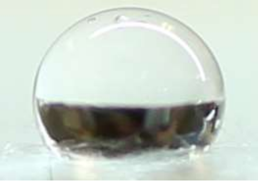 115.84 ± 4.01	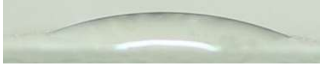 36.04 ± 2.44
	PMMA 2	15 minutes	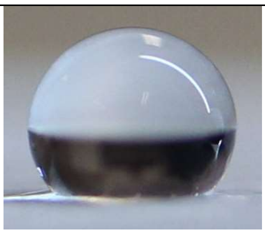 131.90 ± 3.27	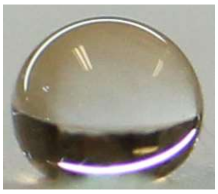 129.07 ± 3.04	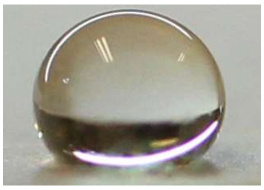 118.24 ± 3.12

		30 minutes	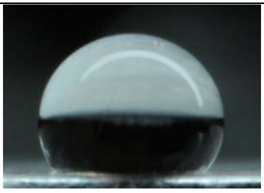<br>125.17 ± 4.20	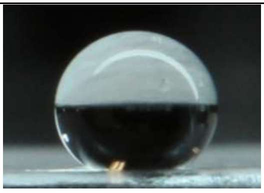<br>126.13 ± 5.78	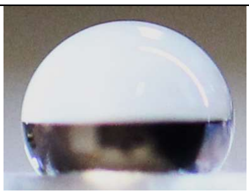<br>119.64 ± 2.28
		45 minutes	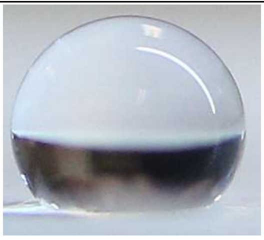<br>130.33 ± 5.00	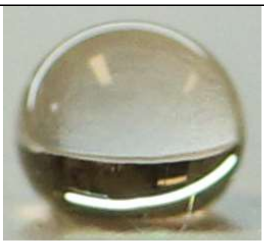<br>126.69 ± 2.75	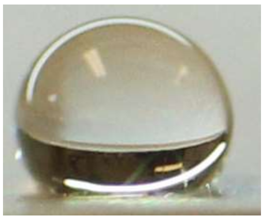<br>118.61 ± 3.13
	**PMMA 3 Fibers**		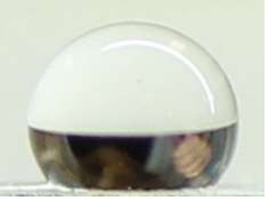<br>118.85 ± 5.83	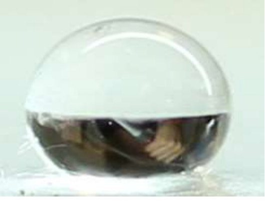<br>114.53 ± 4.55	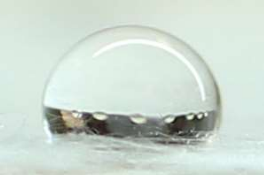<br>116.25 ± 3.95
	**Films**		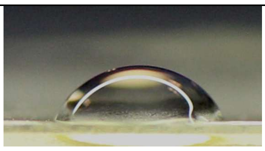<br>69.42 ± 1.91	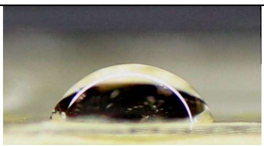<br>70.06 ± 1.11	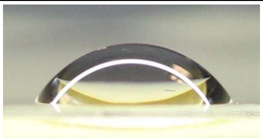<br>46.04 ± 1.60
**PLGA**	**Fibers**		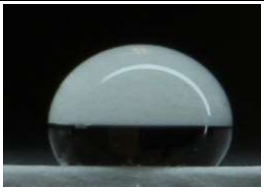<br>121.56 ± 8.49	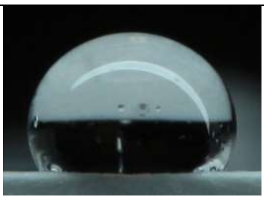<br>118.59 ± 3.32	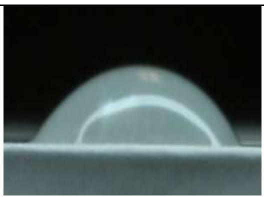<br>57.41 ± 14.24
	**Films**		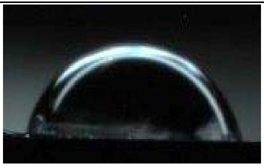<br>72.49 ± 1.41	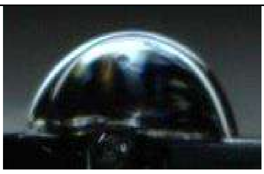<br>69.45 ± 1.41	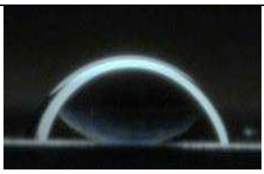<br>60.04 ± 1.99
**PC**	**Fibers**		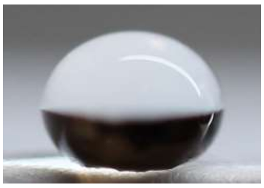<br>145.07 ± 2.63	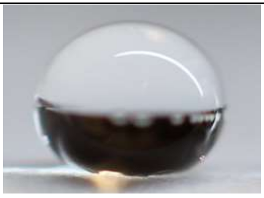<br>143.34 ± 5.22	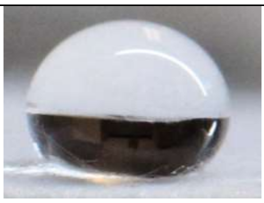<br>127.34 ± 3.26

	**Films**	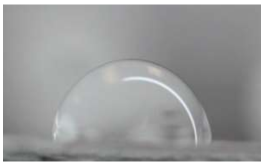<br>91.06 ± 2.84	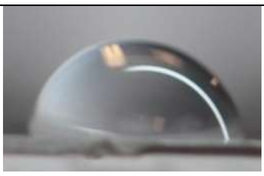<br>74.28 ± 2.96	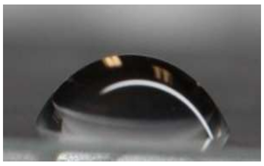<br>60.57 ± 4.19
**PCL**	**Fibers**	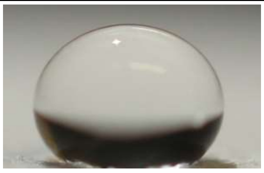<br>129.38 ± 3.29	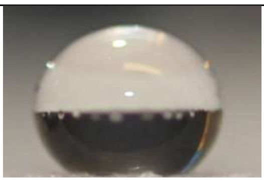<br>119.86 ± 4.36	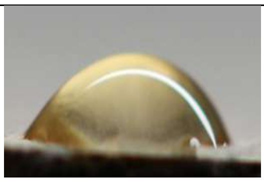<br>46.78 ± 11.2
	**Films**	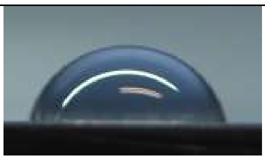<br>73.96 ± 3.97	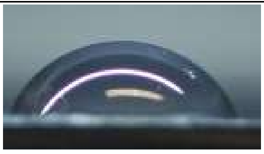<br>74.28 ± 2.96	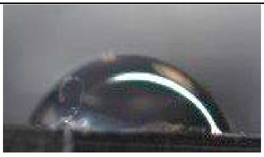<br>48.52 ± 3.33
**PS**	**Fibers**	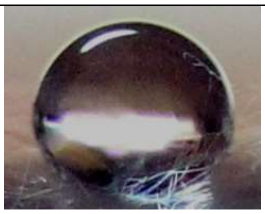<br>124.75 ± 4.92	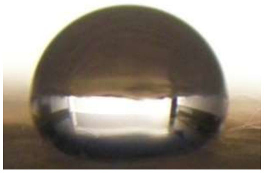<br>132.94 ± 4.21	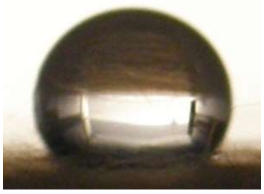<br>117.43 ± 6.32
	**Films**	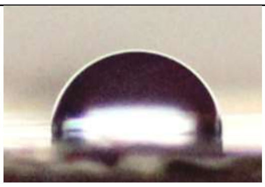<br>82.83 ± 3.04	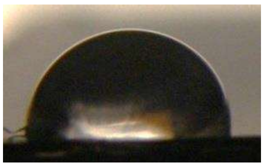<br>80.34 ± 2.37	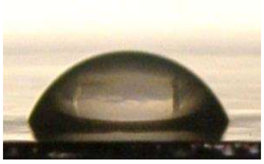<br>67.37 ± 2.28

Table S2. A linear fit to Owens–Wendt plots presented in Figure 4. The intercept and slope were used for the calculation of polar and dispersive components of surface free energy (SFE).

Polymer	PA6	PVDF	PMMA	PLGA	PC	PCL	PS
**Intercept**	0.15160	0.13064	0.15202	0.12659	0.19606	0.18239	0.12722
	±0.01810	±0.06192	±0.05888	±0.02223	±0.00693	±0.00633	±0.03229
**Slope**	0.15150	0.08473	0.11333	0.12519	0.02932	0.08586	0.09537
	±0.01631	±0.05581	±0.05308	±0.02004	±0.00625	±0.00571	±0.02909
**R^2**	0.97710	0.39478	0.64022	0.95004	0.91312	0.99119	0.62064
